# Supplementary material for: Synergistic inhibition of Notch signaling and forced cell cycle re-entry drive Müller glia reprogramming in uninjured mouse retina
Source: eLife. 2026 Sep 17;15:RP111251. doi: 10.7554/eLife.111251 (PMC13585425; doi:10.7554/eLife.111251)
Supplement: Peer review history

# Editor report: eLife Assessment

**DOI:** 10.7554/eLife.111251.3.sa0

**Authors:** Xiaorong Liu

**Affiliations:** University of Virginia United States

**Keywords:** Convincing, Valuable

This study shows that combining forced cell cycle re-entry with *Rbpj* deletion enhances Müller glia dedifferentiation and promotes their conversion into retinal neuron-like cells in the uninjured mouse retina. It provides a **valuable** strategy for improving Müller glia-mediated neurogenesis and advancing regenerative potential in the mammalian retina. Overall, the data are **convincing**. The authors have also addressed concerns regarding Müller glia function, cell survival, and the limitations of neuronal maturation and integration, further strengthening the conclusions of the study.

# Referee report: Reviewer #2 (Public review)

**DOI:** 10.7554/eLife.111251.3.sa1

Summary:

The inability of the mammalian retina to regenerate poses a major clinical challenge. Much has been learned about the regenerative potential of the retina from teleost fish, where Müller glia (MG) are able proliferate and produce new neurons after injury. However, MG do not retain this potential in the mammalian retina. The authors showed previously that that forcing MG to re-enter the cell cycle by downregulating p27 and upregulating cyclin D1 could induce MG to dedifferentiate, but the results were transient, and these cells eventually reverted back to MG and did not form neurons. Here they expand on this to show that in MG, coupling forced cell cycle re-entry with deletion of Rbpj, which inhibits of the transcriptional effects of Notch signaling, induces some MG to proliferate and take on features of multiple cell types, including MG precursor cells, amacrine-like cells, and bipolar-like cells. This work lends valuable insight into the regenerative potential of mammalian MG, particularly when Notch signaling is manipulated.

Strengths:

The major claims of the authors are well-supported. They show convincingly and through multiple methods, including immunostaining, single nucleus RNA sequencing, and in situ hybridization, that coupling notch inhibition with cell cycle re-activation induces the expression of neuronal markers in mammalian MG. The sn-RNA-seq data is particularly valuable in demonstrating the induction of bipolar-cell subtypes. Edu labeling is effective in demonstrating the induction of proliferation, and the long-term viability of the generated neuron-like cells is intriguing.

Comments on revised version:

The authors sufficiently addressed all concerns. I particularly appreciate the additional experiments to demonstrate retinal function, and the edits to the text regarding retinal and cell function and retinal organization.

# Author comment: Author response

**DOI:** 10.7554/eLife.111251.3.sa2

**Authors:** Baoshan Liao, Chengshang Lyu, Yuqing Jiang, Shanggong Liu, Waiho Wong, Jiadong Zhang, Hoyin Tsang, Junxi Xie, Lingxi Chen, Qinrong Zhang, Wenjun Xiong

**Affiliations:** City University of Hong Kong Kowloon Hong Kong; City University of Hong Kong Kowloon Hong Kong; City University of Hong Kong Kowloon Hong Kong; City University of Hong Kong Kowloon Hong Kong; City University of Hong Kong Kowloon Hong Kong; City University of Hong Kong Kowloon Hong Kong; City University of Hong Kong Kowloon Hong Kong; City University of Hong Kong Kowloon Hong Kong; City University of Hong Kong Kowloon Hong Kong; City University of Hong Kong Hong Kong Hong Kong; City University of Hong Kong Kowloon Tong Hong Kong

The following is the authors’ response to the original reviews.

**Public Reviews:**

**Reviewer #1 (Public review):**

Summary:

This study examines Müller glia (MG) reprogramming in the uninjured mouse retina through a combination of Notch signaling inhibition and AAV-induced proliferation. Building on their prior work showing that Cyclin D1 overexpression and p27^Kip1^ knockdown (CCA) promotes MG proliferation with very limited neurogenesis, the authors now demonstrate that Rbpj deletion alone induces a modest degree of MG-to-neuron conversion without proliferation, in agreement with recent work in the field. However, combining Rbpj deletion with CCA-mediated proliferation substantially enhances MG dedifferentiation and the generation of retinal neuron-like cells. Through genetic lineage tracing, histological analyses, and single-cell transcriptomics, the authors provide evidence that MG-derived cells acquire molecular features of bipolar (ON, OFF, and rod bipolar) and amacrine neurons. Most MG-derived cells appear to survive long-term (up to 9 months).

Strengths:

Overall, the study is carefully designed and executed, and the manuscript is clearly written with well-presented figures. While the work does not significantly expand the repertoire of neuronal types generated from mammalian MG beyond what has been previously reported in the field, it provides a valuable and improved strategy for inducing robust MG proliferation and neurogenesis in the mammalian retina.

Weaknesses:

(1) It would be better to include a negative control AAV when evaluating the effect of CCA AAV in the Rbpj KO background. This could help distinguish the specific contribution of the CCA construct from potential effects of intravitreal AAV injection itself, which can induce mild inflammation, known to influence MG reprogramming.

To address this concern, in the revised manuscript we included the result from *Rbpj* KO eyes injected with a negative control AAV (AAV_7m8_-GFAP-GFP) (Fig. S14a). MG reprogramming efficiency, quantified as the proportion of tdT^+^Otx2^+^ cells among total tdT^+^ cells, was then compared between the AAV-GFP–treated and *Rbpj* KO–only eyes. At 4 months post-injection, the percentage of tdT^+^Otx2^+^ cells in the AAV-GFP–treated eyes was comparable to that of Rbpj KO alone (Fig. S14b–c), and substantially lower than in the CCA-treated eyes. Together, these results indicate that the enhanced MG reprogramming observed in the Rbpj KO+CCA group is driven by transgenes expressed rather than by nonspecific effects of AAV or injection.

(2) The extent of MG transduction by the CCA AAV is not clear. As quantifications are normalized to total MG (GFP^+^ or TdTomato^+^) or retinal length, it would be useful to clarify whether near-complete transduction is assumed, or if additional information on transduction efficiency can be provided.

In our previous study (Wu, Liao, et al., 2025, eLife), we have demonstrated that high-dose (4E10vg/injection) AAV7m8 effectively transduced the whole retina, with near-complete MG transduction observed in the vicinity of the injection site, as evidenced by virtually all MG expressing GFP in these regions. In the revised manuscript, we clarified the transduction efficiency in Line 108-110 on Page 5 and Line 625-626 on Page 27.

(3) In Figure S10, the reduced MG proliferation observed in the CCA + Rbpj deletion group could also potentially reflect decreased GFAP promoter activity in dedifferentiated MG following Rbpj deletion. Alternatively, MG-derived cells may be more fragile under these conditions.

We thank the reviewer for these excellent insights. We agree that a down-regulation of GFAP promoter activity following Rbpj-mediated dedifferentiation is a highly plausible explanation for the moderate reduction in proliferation, as lower promoter activity would diminish AAV transgene expression. We have included this possibility in the data interpretation (Line 176-178, page 8). Regarding the alternative possibility of increased cell fragility, we agree that cell death cannot be ruled out, but occasional apoptotic cells over a long period of time are difficult to capture experimentally.

(4) In the CCA + Rbpj deletion condition, do MG undergo single or multiple rounds of cell division?

We have previously demonstrated that MG typically undergo a single round of cell division in wild type mouse retina following CCA treatment (Wu, Liao, et al., 2025, eLife). Given our observation that Rbpj deletion suppresses CCA-induced MG proliferation (Fig. S11), it is unlikely that the addition of *Rbpj* deletion would trigger multiple or continuous rounds of cell division beyond the single-round baseline established by CCA alone. While we did not re-evaluate cell division kinetics in the current study, we reason that CCA similarly drives MG to undergo a single round of division in the *Rbpj* KO context.

(5) What fraction of neuron-like cells (bipolar- and amacrine-like) arises from proliferation versus direct transdifferentiation? Quantification of MG-derived cells expressing neuronal markers (e.g., Otx2, HuC/D), with and without EdU labeling, would help distinguish these mechanisms.

The percentages of MG-derived cells expressing neuronal markers with and without EdU labeling, were shown in Fig 3d-e and Fig S19d-e. In the Rbpj KO-only group, neuron-like cells arise exclusively through direct transdifferentiation without cell division, as no EdU incorporation was detected in Rbpj-deficient MG. In this group, a small fraction of MG-derived cells expressed the neuronal marker Otx2 or HuC/D (Fig 3e, Fig S19e). In contrast, the Rbpj KO+CCA group achieved a substantially higher neurogenesis rate, with a significant proportion of Otx2+ or HuC/D+ MG-derived cells also being EdU+ (Fig. 3d, Fig. S19d), indicating that they arose through de novo neurogenesis. By subtracting the contribution of direct transdifferentiation observed in the Rbpj KO-only group, we estimate that majority of MG-derived neuron-like cells in the Rbpj KO+CCA group were generated through proliferation-mediated de novo neurogenesis.

(6) In Figure S18a, the authors state that "while the neuron-like clusters were best classified as BC-like and AC-like based on their distinct marker gene expression, they also exhibited mixed expression of genes associated with other retinal neuronal types, including RGC markers (e.g., Tubb3, Myt1l, Grin1) and photoreceptor markers (e.g., Crx, Prom1, Epha10, Gucy2e, Scg3) (Fig. S18a), suggesting that the regenerated cells exist in a hybrid state" and "MG derived neuron like cells also expressed genes characteristic of RGCs and photoreceptors, indicating enhanced lineage". However, many of these genes are not specific to RGCs or photoreceptors and are instead broadly expressed in retinal neurons or enriched in bipolar/amacrine populations. Therefore, it is unclear whether these cells exhibit hybrid RGC or photoreceptor identity.

We thank the reviewer for this insightful comment and for pointing out the need for greater precision in our terminology regarding these markers. While individual markers may lack absolute, 100% cell-type exclusivity, genes such as *Tubb3* and *Gucy2e* serve as widely accepted lineage-associated genes that characterize RGC and photoreceptor programs, respectively (Soto et al., 2008; Sato et al., 2018; Sotani et al., 2024). We have revised the manuscript to replace terms "RGC-specific genes" and "photoreceptor-specific genes" with "RGC signature genes" and "photoreceptor signature genes", respectively. Furthermore, these RGC- and photoreceptor-signature genes are co-expressed across the entire Otx2+ MG population rather than being segregated into distinct, specialized subpopulations (Fig. 4d, Fig. S20). This uniform distribution indicates that these cells possess a hybrid transcriptional program that concurrently incorporates elements of both RGC and photoreceptor identities.

(7) The authors provide a thorough molecular characterization of MG-derived cells through immunostaining and single-cell sequencing. However, their morphological features, synaptic connectivity (e.g., synaptic marker expression), and electrophysiological properties remain largely uncharacterized. While these experiments may be technically challenging, this limitation should be discussed.

We agree with the reviewer that characterizing the precise morphological features, synaptic connectivity, and electrophysiological properties of MG-derived cells is a crucial step for any neuronal regeneration study, and we acknowledge that this represents an important limitation of our current study.

As demonstrated by snRNA-seq data, the MG-derived neuron-like cells exhibit an incompletely mature state, characterized by hybrid transcriptomic signatures. By immunostaining, we did not observe any MG-derived cells with photoreceptor outer segment or typical RGC morphology. Therefore, it is highly likely that these cells have not established functional synaptic connectivity or acquired mature electrophysiological properties. Performing functional or circuitry assessments at this stage would be premature.

We have added a comprehensive discussion regarding this limitation, along with future directions for long-term functional validation, in the revised manuscript (Line 502-515 on Page 22).

(8) The conclusion that CCA + Rbpj deletion induces neurogenesis without compromising MG supportive functions or retinal homeostasis appears somewhat oversold. This claim is primarily based on gross retinal morphology and ZO-1 staining. Given the extent of MG dedifferentiation and ectopic cell generation in the ONL and INL, it is likely that retinal function is affected. Functional assessments (e.g., ERG) would be required to support this conclusion. The authors should consider tempering this statement.

To address the concern raised by the reviewer, we performed electroretinography (ERG) to evaluate both scotopic and photopic retinal function in the Rbpj KO+CCA-treated eyes compared to contralateral untreated controls (Supplementary figure S23e-h). In addition, we conducted optomotor response testing to assess whether visual behavior is affected following treatment (Supplementary Figure S23d). The results demonstrate that combined Rbpj KO and CCA treatment achieves neurogenesis without compromising retinal function.

(9) Regarding the mechanism by which CCA-induced proliferation enhances MG reprogramming in the Rbpj knockout background, one plausible explanation is that chromatin states (e.g., histone modifications and DNA methylation) are transiently reset during DNA replication and cell division. While this alone may be insufficient to activate neurogenic programs, it could synergize with Rbpj deletion to allow neurogenic transcription factors (such as Ascl1, Otx2, NeuroD1, and NeuroD2) to access previously inaccessible chromatin regions, thereby promoting MG reprogramming.

We thank the reviewer for the insightful suggestion on the model, which aligns well with our experimental findings. Our snATAC-seq data demonstrate that CCA-induced proliferation broadly increases chromatin accessibility at key neurogenic loci, including Neurod2, Dll1, and Otx2, in active MG compared to resting MG (Figure 6f–h). This chromatin remodeling alone is insufficient to drive neurogenesis, as CCA-only treated MG largely revert to a quiescent glial state. However, when combined with Rbpj deletion, which derepresses downstream neurogenic transcription factors such as Ascl1 and Neurog2 by relieving Notch-mediated transcriptional repression, these newly accessible chromatin regions can be effectively occupied and activated by the available neurogenic factors. The concept that cell division facilitates epigenetic resetting to enhance reprogramming efficiency is well established in the somatic cell reprogramming field, where proliferation rate is directly proportional to reprogramming success by promoting the erasure of lineage-restrictive epigenetic marks and the re-establishment of new transcriptional circuits. In the revised manuscript, we incorporated this mechanistic discussion to provide a more comprehensive interpretation of how proliferation and Notch inhibition converge to promote MG neurogenesis in Line 462-477 on Page 20-21.

**Reviewer #2 (Public review):**

Summary:

The inability of the mammalian retina to regenerate poses a major clinical challenge. Much has been learned about the regenerative potential of the retina from teleost fish, where Müller glia (MG) are able to proliferate and produce new neurons after injury. However, MG do not retain this potential in the mammalian retina. The authors showed previously that forcing MG to re-enter the cell cycle by downregulating p27 and upregulating cyclin D1 could induce MG to dedifferentiate, but the results were transient, and these cells eventually reverted back to MG and did not form neurons. Here, they expand on this to show that in MG, coupling forced cell cycle re-entry with deletion of Rbpj, which inhibits the transcriptional effects of Notch signaling, induces some MG to proliferate and take on features of multiple cell types, including MG precursor cells, amacrine-like cells, and bipolar-like cells. This work lends valuable insight into the regenerative potential of mammalian MG, particularly when Notch signaling is manipulated.

Strengths:

The major claims of the authors are well-supported. They show convincingly - and through multiple methods including immunostaining, single-nucleus RNA sequencing, and in situ hybridization - that coupling notch inhibition with cell cycle reactivation induces the expression of neuronal markers in mammalian MG. The snRNA-seq data are particularly valuable in demonstrating the induction of bipolar-cell subtypes. Edu labeling is effective in demonstrating the induction of proliferation, and the long-term viability of the generated neuron-like cells is intriguing.

Weaknesses:

Whether the newly generated neurons are functionally integrated remains unclear, and the effect of the manipulation on the function of the retina was not tested. Imaging data suggests that many of the newly generated neurons persist for months, but often appear mislocalized. It is also not clear if the manipulation of MG affects long-term MG function. Cell death was not evaluated, and although the authors evaluated the long-term effect on tight junctions, this data was not quantified, and further analysis on morphology or function was not done. Control eyes were untreated, not vehicle-injected.

**Recommendations for the authors:**

**Reviewer #1 (Recommendations for the authors):**

(1) The transgenic line may be Glast-CreERT, not Glast-CreERT2.

We appreciate the reviewer for bringing this to our attention. The formal allele symbol for this transgenic line is Tg(Slc1a3-cre/ERT)1Nat, while this strain is generically classified as "Cre/ERT2" by the Jackson Laboratory. Some published studies referred to this line as Glast-CreERT and others as Glast-CreERT2. To maintain consistency with the formal allele symbol, we have adopted "Glast-CreERT" throughout the revised manuscript.

(2) For snATAC data in Figure 6 e,f, and Figure 19b. It is most likely gene activity, not gene expression, since these are snATAC, not snRNA data.

For this inaccurate terminology, we have corrected all relevant figure labels and associated text in the revised manuscript to clearly state "gene activity" instead of "gene expression."

(3) Some text in Figure 6 is a bit too small to read.

We have increased the font size of the text elements in Figure 6 to ensure readability and have also reviewed all other figures for consistency. Revised figures with improved legibility have been included in the updated manuscript.

**Reviewer #2 (Recommendations for the authors):**

(1) There are multiple instances where further elaboration of methods or tools in the test would improve readability and comprehension by a broader audience. It would be helpful to (early, often, and clearly) explain precisely which cell types are labeled in your mouse line and how. Someone unfamiliar with the mouse line may struggle to understand what is labeled by the tdT or GFP. Likewise, it would help to consistently define what cell types are labeled by tdT+ vs. Sox9+, tdT+, etc.

We have added a clear and detailed description of the mouse lines and labeling strategy early in the Results section, specifying which cell types are labeled by tdT and GFP and how the labeling is achieved. We have also ensured that the definitions of cell type identifiers (e.g., tdT^+^ for MG-derived cells, Sox9^+^/tdT^+^ for MG remaining in a glial state) are consistently stated upon first use and maintained throughout the manuscript to improve readability for a broader audience. In addition, we added headings for the quantification graphs to improve readability in all quantification figures.

(2) It is unclear what the difference is between Figure 1c and S1c, and these should be quantified as the % of positive cells, as described in the text.

We have removed Figure S1c and moved Figure 1c to supplementary figure 1. The MG labeled by EdU and Sox9 or Otx2 were quantified as % of the EdU+ MG.

(3) S2e: Clarify what pixel level means, is this pixel intensity?

Yes, "pixel level" in Figure S2e refers to pixel intensity. We apologize for the ambiguous wording and replaced "pixel level" with "pixel intensity" in the revised figure legend to ensure clarity.

(4) Figure 2: In the magnified image of the GFP+, Sox9- cell, the GFP is also very faint. Could these cells be dying? Analysis of the expression profile of these cells (or ruling out apoptosis) would better support a dedifferentiation argument.

The faint GFP signal observed in GFP^+^ Sox9^-^ cells is a sign of ongoing dedifferentiation rather than cell death. This is likely due to chromatin remodeling during reprogramming. A similar decrease in reporter signal intensity during MG dedifferentiation has been previously reported by Le et al. 2024, 2025, supporting the interpretation that reduced fluorescence is a characteristic feature of this process. It is possible that a small fraction of GFP^+^ Sox9^-^ cells may undergo cell death over an extended period, which would be difficult to detect using apoptosis assays. Our long-term survival experiments demonstrate that more than 80% of MG-derived neuron-like cells survive for at least 9 months following treatment (Figure 7), indicating that majority of these cells are viable. The discussion is included in line 112-114 on page 5.

(5) Figure 3: The Crx labeling appears everywhere except the identified cell. This seems the opposite of the point you are making.

Crx signal of the MG-derived cell (tdT^+^ Crx^+^), which is pointed out by arrowhead, is in a ring-like pattern. This pattern is consistent with the euchromatin region in inverted nucleus of rod. Crx labeling appears in other cells in the image as Crx is highly expressed in native photoreceptors.

(6) I think it would be nice to address, in the discussion, the apparent disorganization and mislocalization of cells in the long-term images.

We thank the reviewer for highlighting this critical observation. During retinal development, precise laminar positioning of neurons is guided by a coordinated interplay of cell-intrinsic transcriptional programs and extrinsic cues including cell adhesion molecules, guidance factors, and interactions with neighboring cells. In the adult retina, many of these developmental cues are no longer present or active, which likely contributes to the failure of MG-derived neurons to migrate to their appropriate laminar positions. Interestingly, the vast majority of our divided MG cells remained localized within the outer nuclear layer (ONL). Because the ONL is the physiological location of photoreceptors, this preferential position could serve as an advantageous baseline layout for driving targeted photoreceptor differentiation in future work. To address reviewer’s feedback, we have expanded our discussion section (Line 543-559, page 23-34) to cover the mechanisms underlying this structural disorganization and its downstream implications for functional circuit integration.

(7) I'm not convinced that ZO1 alone is sufficient to suggest MG function normally or that retinal homeostasis is maintained. I suggest tempering that conclusion in the text.

For the revision, we have performed additional experiments to address this concern. The optical coherence tomography (OCT) images revealed that retinal layer organization and ONL thickness were comparable among the uninjected eyes, GFP AAV-injected control eyes, and CCA-treated eyes, demonstrating that overall retinal architecture was well-preserved (Fig. S23a–c). Optomotor response testing revealed no significant differences in visual acuity across groups, suggesting that visual function remained intact (Fig. S23d). Furthermore, electroretinography (ERG) demonstrated that scotopic and photopic a- and b-wave amplitudes were unaffected by the treatment, confirming that light responses from photoreceptor and inner retinal neuron were preserved (Fig. S23e–h). Taken together, these findings demonstrate that combined Rbpj KO and CCA treatment achieves neurogenesis without compromising retinal structure and functional visual circuitry.
